# LLMs in Medical Education for Autism Caregivers: A Comparative Evaluation of Accuracy, Readability, Actionability, and Neurodiversity-Affirming Language

**DOI:** 10.3390/healthcare14142137

**Published:** 2026-07-16

**Authors:** Shahid Akhtar Akhund, Asma Alsaleh, Naheed Haroon Kazi, Bheemsain Rajpal, Shahmina Naz, Shoukat Ali Arain

**Affiliations:** 1 Department of Medical Education and Anatomy and Genetics, College of Medicine, Alfaisal University, Riyadh 11533, Saudi Arabia; sakhund@alfaisal.edu; 2 Autism Center of Excellence (ACE), Amr Ibn Alaas St., Ulaishah, Riyadh 11564, Saudi Arabia; alsaleh.asma@gmail.com; 3 Muhammad Medical College Hospital, Ibn-e-Sina University, Mirpur Khas 69000, Pakistan; naheedharoonkazikazi412@gmail.com; 4 Ipswich Hospital, Queensland Health, Ipswich, QLD 4305, Australia; bheemsain.rajpal@health.qld.gov.au; 5 Department of Anatomy and Genetics, College of Medicine, Alfaisal University, Riyadh 11533, Saudi Arabia; snaz@alfaisal.edu; 6 Department of Pathology, College of Medicine, Alfaisal University, Riyadh 11533, Saudi Arabia

**Keywords:** autism spectrum disorder, large language models, PEMAT-P, FKGL, SMOG, Saudi Arabia, caregiver education, neurodiversity-affirming language, health literacy, ChatGPT, Gemini, DeepSeek

## Abstract

**Highlights:**

**What are the main findings?**

- Based on 24 selected questions from Saudi Arabian context comparing three LLMs during short single time window, Google Gemini 1.5 Pro achieved the highest scientific accuracy, significantly outperforming DeepSeek (*p* = 0.003, medium effect), though not ChatGPT, under notable ceiling-effect conditions; OpenAI ChatGPT (GPT-4o) provided the highest understandability, and DeepSeek-V3 yielded the highest actionability.
- All three tested LLMs failed to meet the AHRQ 80% actionability benchmark and fell short of recommended readability thresholds (produced the text above FKGL 6 and SMOG 8 threshold levels), indicating grade levels are too complex for general caregiver audiences) on English outputs.Accuracy errors across all models consistently clustered on localized, Saudi-specific epidemiology, genetic risk, and financial/healthcare service inquiries.

**What are the implications of the main findings?**

- Current commercial LLMs require extensive plain-language adaptation and precise cultural/geographical customization before they can safely serve as localized health information tools.
- Clinicians must proactively guide family caregivers on navigating AI outputs, explicitly warning them about inaccuracies in country-specific economic, epidemiological, and healthcare service queries.

**Abstract:**

**Background:** Family caregivers of children with autism spectrum disorder (ASD) increasingly utilize large language models (LLMs) for health information. This study presents a systematic comparative evaluation of three widely used LLMs as localized ASD health information tools in Saudi Arabia. **Methods:** Twenty-four clinically validated, caregiver-oriented questions were posed to Google Gemini 1.5 Pro, OpenAI ChatGPT (GPT-4o), and DeepSeek-V3 using a standardized prompt. Three expert raters independently evaluated responses across four dimensions: scientific accuracy, PEMAT-P understandability, PEMAT-P actionability, and neurodiversity (ND)-affirming language. Readability was assessed via Flesch–Kincaid Grade Level (FKGL) and SMOG indices. Non-parametric Kruskal–Wallis tests with post hoc Mann–Whitney U comparisons and one-sample t-tests were applied. **Results:** Gemini achieved the highest mean accuracy (2.96/3.00), significantly outperforming DeepSeek (*p* = 0.003, r = −0.37). Accuracy failures across all LLMs clustered on regional epidemiological, genetic risk, and financial inquiries. ChatGPT achieved significantly higher understandability than Gemini (*p* < 0.001, r = 0.55), while DeepSeek achieved significantly higher actionability than Gemini (*p* < 0.001, r = 0.59). However, all three LLM scores fell short of the Agency for Healthcare Research and Quality (AHRQ) 80% actionability benchmark (all *p* < 0.001). All LLMs exceeded patient education readability benchmarks (FKGL ≤ 6, SMOG ≤ 8; all *p* < 0.001); ChatGPT was the most readable (FKGL = 7.86; SMOG = 9.97) and Gemini the most complex. No model differed significantly on ND-affirming language, defaulting to a mixed medical-affirming register. **Conclusions:** Evaluated LLMs demonstrated distinct, specialized strengths: Gemini was the most accurate, ChatGPT the most readable, and DeepSeek the most actionable. Importantly, all models failed to meet established consumer education standards for readability and actionability. LLMs require extensive plain-language adaptation and cultural customization. Clinicians must guide families on navigating LLM outputs, particularly concerning country-specific epidemiological, economic, and healthcare service queries.

## 1. Introduction

### 1.1. Autism Spectrum Disorder: Burden, Complexity, and the Caregiver Imperative

Autism spectrum disorder (ASD) is a complex, lifelong neurodevelopmental condition characterized by persistent differences in social communication and interaction, restricted and repetitive patterns of behavior, and distinct sensory processing profiles [1,2]. The DSM-5 consolidated previously distinct ASD subtypes into a unified spectrum, reflecting the wide heterogeneity in clinical presentation and support needs [3]. Early behavioral signs are often detectable before 12 months, yet the global median age at formal diagnosis remains approximately 60 months, representing a critical window of missed early intervention [4]. Global ASD prevalence is estimated at approximately 1%, though rates in Saudi Arabia are estimated at approximately 1 in every 180 children, which are likely underestimates given documented regional barriers to diagnosis and limited specialist availability [2,5,6,7]. The range could not be precisely established due to limited population-based epidemiological studies. For context, the World Health Organization estimates a global prevalence of approximately 1 in 100 children [8].

Co-occurring behavioral or emotional challenges are common among autistic children, and with appropriate support, many children manage these effectively [9]. ASD’s etiology is multifactorial, involving synaptic, transcriptional, and chromatin gene disruptions alongside prenatal environmental risk factors [10,11]. These neurobiological underpinnings produce a heterogeneous clinical picture that challenges families and clinicians alike. Families constitute the primary support system across the lifespan yet frequently face substantial informational and systemic barriers; caregiver stress is significantly elevated relative to parents of neurotypical children. Studies in Saudi Arabia have documented poor parental ASD knowledge, particularly among fathers with lower educational attainment [6,7].

### 1.2. Large Language Model Families in Healthcare: Promise, Peril and Differences

The rapid proliferation of AI-driven large language models (LLMs), including OpenAI’s ChatGPT, Google’s Gemini, and DeepSeek, has fundamentally transformed public access to health information [12]. In Saudi Arabia, where 92.6% of the population accesses health information online and 76.1% through social media, adoption of LLMs is accelerating. Parents of children with ASD are particularly active consumers of digital health content, frequently turning to online resources to supplement limited access to specialist services [13].

Despite growing reliance, LLMs have significant limitations as health information tools: susceptibility to hallucinations, lack of real-time knowledge updates, training data biases, and an absence of cultural contextualization [14]. In ASD, a domain with a documented history of vaccine-related and etiological misinformation, these limitations carry particular clinical risk [15]. The quality of online ASD health information for caregivers is variable, and it is not established whether LLM-generated responses represent an improvement on this baseline. Critically, no comparative evaluation of LLMs as ASD caregiver information tools has been conducted using validated, multi-dimensional quality instruments, and no evaluation has been conducted in an Arab or Middle Eastern context.

Large language models (LLMs) have been evaluated for medical knowledge accuracy in Saudi Arabia [16] and Arabic healthcare communication [17], and separately for autism caregiver questions [18,19,20], and PEMAT has been applied to LLM-generated patient materials elsewhere [21]. However, no prior study jointly applies PEMAT-P understandability/actionability, dual readability metrics (FKGL, SMOG), and neurodiversity-affirming language scoring to autism caregiver content across ChatGPT, Gemini, and DeepSeek within a Middle Eastern context, distinguishing this study from each isolated strand of prior work.

The three evaluated systems differ substantially in architecture, training, and alignment, which plausibly explains their divergent behavior on accuracy, formatting, and actionability. Gemini 1.5 Pro is a Mixture-of-Experts (MoE) Transformer with native multimodal fusion and Multi-Query Attention, optimized for long-context retrieval and reasoning across text, image, audio, and video inputs [22]. GPT-4o is OpenAI’s omni-modal model, aligned through supervised fine-tuning and Reinforcement Learning from Human Feedback (RLHF) with Proximal Policy Optimization (PPO), producing consistently structured, human-preference-tuned chat formatting [23]. DeepSeek-V3 uses a distinct MoE architecture with Multi-Head Latent Attention (MLA) for efficient inference, an auxiliary-loss-free load-balancing strategy, and a multi-token prediction training objective, with post-training alignment via Group Relative Policy Optimization (GRPO) rather than standard PPO-based RLHF [24]. As Bhati et al. (2026) note in their cross-family survey, models emphasizing attention-efficiency optimizations (e.g., DeepSeek’s MLA, Gemini’s MQA) tend to pair these with lighter alignment pipelines, whereas models with deeper RLHF/PPO alignment (e.g., GPT-4o) trade some efficiency for more consistent, human-preferred output formatting—a pattern consistent with the accuracy, structure, and actionability differences we observed across models [25].

### 1.3. Theoretical Framework

This study is grounded in health literacy theory, operationalized through the Patient Education Materials Assessment Tool for Printable Materials (PEMAT-P) [26], which provides a validated framework for evaluating whether health information is understandable and actionable for lay audiences. It additionally draws on the information quality framework of consumer health informatics, which recognizes that effective health information must satisfy three interrelated criteria: scientific accuracy, linguistic accessibility, and neurodiversity-affirming framing. Readability is operationalized using the Flesch–Kincaid Grade Level (FKGL) and the Simple Measure of Gobbledygook (SMOG) index, both endorsed by the NIH for patient education material evaluation.

The language used to describe autism carries clinical and psychological weight for caregivers. Deficit-based framing (e.g., describing autistic traits primarily as impairments or deficits to be corrected) has been criticized as stigmatizing and inconsistent with autistic self-advocacy perspectives, whereas neurodiversity-affirming language frames autism as a form of natural human variation while still acknowledging support needs [27]. Prior work assessing online autism information has similarly highlighted the coexistence of medicalized and affirming registers in caregiver-facing content, and the consequences this has for trust and engagement [28]. Because caregivers increasingly turn to LLMs for this kind of information, whether these models default to deficit-based or affirming language is a direct extension of this literature, motivating RQ4 below.

### 1.4. Research Questions and Aims

This study addresses four primary research questions:

- RQ1. How do Gemini, ChatGPT, and DeepSeek compare in scientific accuracy when responding to common ASD caregiver questions?
- RQ2. To what extent are LLM-generated ASD responses understandable and actionable for lay caregivers, as assessed by the PEMAT-P?
- RQ3. What is the reading grade level of LLM responses, measured by FKGL and SMOG, relative to patient education benchmarks?
- RQ4. Do LLMs employ ND-affirming language, or do they default to deficit-based, medicalized framing?

## 2. Materials and Methods

### 2.1. Study Design, Setting, and Ethics

This cross-sectional, comparative evaluation study was conducted at the College of Medicine, Alfaisal University, Riyadh, Kingdom of Saudi Arabia, from April to August 2025. The study evaluated publicly generated output from commercially available AI platforms and involved no human participants, patient-identifiable data, animal subjects, or clinical interventions. Formal ethics committee review was not required in accordance with international guidance for this type of health informatics research [29].

### 2.2. Question Bank Development

A 24-question bank was developed through a structured multi-stage process. An initial pool of 48 candidate questions was compiled from consultations with healthcare providers, published ASD caregiver brochures from the Autism Society of America and the Saudi Autism Society, and ASD advocacy website FAQs. An expert validation panel of two senior consultant pediatricians and clinical nursing staff independently ranked all 48 questions on frequency of clinical occurrence and clinical significance; the 25 highest-scoring questions were selected and refined through group consensus to a final bank of 24 questions covering the full range of ASD caregiver concerns from etiology and diagnosis through management, school placement, and long-term prognosis (Supplementary File (S6)). This was a single-round expert consensus process rather than a formal Delphi procedure (which would involve iterative, anonymous rounds with controlled feedback), and it did not include caregiver or parent raters.

### 2.3. LLM Selection and Response Generation

Three LLMs were selected for this analysis: Gemini 1.5 Pro (Google LLC, Mountain View, CA, USA), ChatGPT running the GPT-4o architecture (OpenAI, San Francisco, CA, USA), and DeepSeek-V3 (DeepSeek, Hangzhou, China). Selection criteria were high global user volumes, documented use in comparable health evaluation studies, and free public accessibility [19]. A uniform parent-friendly prompt was applied to all LLMs for every question, which stated that “We are the parents of a child who is possibly suffering from autism; answer our following question precisely in a way that a common person can understand”. The uniform, parent-friendly prompt was used deliberately, without system-role assignment, prompt optimization, or deterministic decoding parameters (e.g., low temperature), in order to reflect the way a typical caregiver would realistically interact with these models via consumer chatbot interfaces, rather than expert or API-level usage. Each question was entered into a fresh dialog session to eliminate conversational memory effects. Only one response was generated for each question by each LLM used. All responses were generated within a two-month window and stored verbatim. All LLMs were accessed via consumer web interfaces (chat.google.com/Gemini 1.5 Pro; chat.openai.com/GPT-4o; chat.deepseek.com/DeepSeek-V3) using free accounts, from April to May 2025. No API was used; decoding parameters were not configurable. Web search was disabled where available. LLMs answered based on their internal knowledge. One response per question-model was captured without regeneration. The English interface was used throughout the sessions.

### 2.4. Evaluation Framework

Each response was independently evaluated across four validated dimensions:

- Scientific accuracy (Criteria 1): Three-point ordinal scale (1 = completely incorrect/unclear/not concise; 3 = completely correct, clear, and concise), adapted from the Information Quality Grading framework [29].
- PEMAT-P understandability (Criteria 2a): Items 1–12 of the PEMAT-P [26] were scored dichotomously and expressed as a percentage; ≥80% is the Agency for Healthcare Research and Quality (AHRQ) acceptable threshold. Items 13–19 (visual aids) and 25–26 (visual actionability) were scored Not Applicable per PEMAT-P protocol, as plain-text LLM responses contain no images or diagrams; these were excluded from denominators.
- PEMAT-P actionability (Criteria 2b): Items 20–24 of the PEMAT-P were scored and expressed equivalently; ≥80% is the AHRQ acceptable threshold.
- ND-affirming language (Criteria 3): Three-point ordinal scale (1 = predominantly medical/deficit-based language; 3 = predominantly ND-affirming language), Scores were operationalized as: 1 = pathologizing/deficit-based language (e.g., “suffering from autism,” “cure,” “abnormal”); 2 = mixed clinical and affirming register; 3 = predominantly ND-affirming, strengths-based language (adapted from Bottema-Beutel et al., 2021; Kenny et al., 2016) [27,28]. Rating examples were provided to raters at calibration. This dimension reflects evidence that language framing influences parental coping and treatment engagement.
- Readability (Criteria 4): Flesch–Kincaid Grade Level (FKGL; target ≤ 6, AMA/NIH) and SMOG index (target ≤ 8, NIH), computed algorithmically from verbatim response text. Full formulae are provided in the Supplementary File (S4).

### 2.5. Raters and Blinding

Three raters independently evaluated all responses: A.A. (ASD education support specialist), N.H.K. (Pediatrician, ASD family support specialist), and B.R. (Senior Pediatrician and public health communication specialist). The raters were blinded to one another’s assessments and to LLM identity throughout scoring. Data custodianship was restricted to two authors (S.A.Ak. and S.A.Ar.), who did not contribute evaluation scores. The individual question-level scores from all three raters across all criteria and all three LLMs can be shared with readers on request.

### 2.6. Statistical Analysis

All analyses were performed using IBM SPSS Statistics version 30.0 (IBM Corp., Armonk, NY, USA). Inter-rater reliability across the three human raters was quantified using the intraclass correlation coefficient, ICC (2, 1) (two-way random, absolute agreement, single measure). Normality was assessed using the Shapiro–Wilk test. Non-parametric Kruskal–Wallis H tests compared the three LLMs on all ordinal and non-normally distributed outcomes, followed by Bonferroni-corrected Mann–Whitney U post hoc comparisons. Bonferroni correction was applied within each family of pairwise comparisons (e.g., across models for accuracy, understandability, actionability, and readability separately), controlling family-wise error at α = 0.017 within each family. Correction was not applied across families; the cumulative Type I error risk across all statistical tests conducted in this study was therefore not controlled and should be considered when interpreting the overall pattern of significant findings. Effect sizes are reported as rank-biserial correlation (r). One-sample t-tests compared normally distributed FKGL and SMOG scores against published benchmarks. One-sample Wilcoxon signed-rank tests compared PEMAT-P percentages against the 80% benchmark.

## 3. Results

### 3.1. Inter-Rater Reliability

Inter-rater reliability analysis, presented in Table 1, was conducted to assess the consistency among the three raters using ICC (2, 1) estimates. Agreement was excellent and perfect for Gemini (ICC = 1.000) and ChatGPT (ICC = 1.000) on scientific accuracy, reflecting a ceiling effect in which all three raters assigned identical scores for 24 of 24 questions. DeepSeek showed moderate agreement on this criterion (ICC = 0.631, 95% CI [0.404, 0.804]), with disagreement concentrated on questions about prevalence (Q03), treatment cost (Q17), and long-term management. ChatGPT achieved excellent reliability on both PEMAT-P subscales (understandability: ICC = 0.947; actionability: ICC = 0.948); Gemini showed poor understandability reliability (ICC = 0.446) and good actionability reliability (ICC = 0.839); DeepSeek showed moderate reliability on both PEMAT-P subscales. For ND-affirming language, reliability ranged from moderate for Gemini (ICC = 0.635) to excellent for DeepSeek (ICC = 0.912).

### 3.2. Scientific Accuracy (RQ1)

Accuracy scores were exceptionally good and non-normally distributed for all LLMs (all W < 0.75, *p* < 0.001). Gemini achieved the highest mean accuracy (M = 2.96, SD = 0.20, Mdn = 3.00) across 24 questions, followed by ChatGPT (M = 2.79, SD = 0.51) and DeepSeek (M = 2.64, SD = 0.47). The Kruskal–Wallis test indicated a significant overall difference, H(2) = 9.23, *p* = 0.010. Post hoc comparisons showed Gemini significantly outperformed DeepSeek (U = 394.0, *p* = 0.003, r = −0.37, medium effect); Gemini vs. ChatGPT (*p* = 0.161) and ChatGPT vs. DeepSeek (*p* = 0.119) were non-significant. Accuracy failures across all LLMs clustered on questions about ASD prevalence in Saudi Arabia (Q03), genetic risk (Q07), and treatment cost (Q17).

### 3.3. PEMAT-P Understandability and Actionability (RQ2)

Understandability scores were non-normally distributed (all *p* < 0.01). ChatGPT achieved the highest mean (M = 81.94%, SD = 11.05%), followed by DeepSeek (M = 81.36%, SD = 7.02%) and Gemini (M = 79.50%, SD = 4.90%). Kruskal–Wallis analysis indicated a significant overall difference, H(2) = 9.03, *p* = 0.011. ChatGPT significantly outperformed Gemini (U = 129.0, *p* < 0.001, r = 0.55, large effect); other pairwise comparisons were non-significant. Importantly, none of the three LLMs fell significantly below the 80% AHRQ understandability benchmark (all *p* > 0.05), a favorable finding indicating that understandability was broadly acceptable. This contrasts with actionability; all LLMs failed to reach the 80% threshold, *p* < 0.001. Actionability was clearly below the recommended standards.

Actionability scores (all *p* < 0.05 for non-normality) were substantially lower than understandability for all LLMs: Gemini M = 51.11% (SD = 11.06%), ChatGPT M = 56.11% (SD = 17.57%), and DeepSeek M = 65.83% (SD = 13.09%). One-sample Wilcoxon tests confirmed all three LLMs scored significantly below the 80% actionability benchmark (all *p* < 0.001). Kruskal–Wallis analysis was significant, H(2) = 12.06, *p* = 0.002; DeepSeek significantly outperformed Gemini (U = 119.5, *p* < 0.001, r = 0.59, large effect).

### 3.4. Readability (RQ3)

FKGL and SMOG scores were normally distributed for all LLMs (all W > 0.94, all *p* > 0.20). The readability scores were clearly above the recommended benchmark. All LLMs significantly exceeded the FKGL ≤ 6 benchmark: Gemini t(23) = 8.53, *p* < 0.001, d = 1.74; ChatGPT t(23) = 4.90, *p* < 0.001, d = 1.00; DeepSeek t(23) = 8.99, *p* < 0.001, d = 1.83. All LLMs also significantly exceeded the SMOG ≤8 benchmark (all *p* < 0.001, all d > 1.40). Kruskal–Wallis tests showed significant between-LLM differences in FKGL [H(2) = 13.56, *p* = 0.001] and SMOG [H(2) = 21.26, *p* < 0.001]. Gemini was significantly more complex than ChatGPT on both indices (FKGL: r = −0.57; SMOG: r = −0.70, both *p* < 0.001). Full descriptive statistics and pairwise comparison results are presented in Table 2 and Table 3.

### 3.5. Neurodiversity-Affirming Language (RQ4)

Language scores were non-normally distributed for all LLMs (all *p* < 0.001). Mean scores were comparable: Gemini M = 2.12 (SD = 0.29), ChatGPT M = 2.14 (SD = 0.32), DeepSeek M = 2.21 (SD = 0.55). The Kruskal–Wallis test showed no significant difference, H(2) = 0.35, *p* = 0.838. All pairwise comparisons were non-significant (all *p* > 0.50). All three LLMs predominantly defaulted to a mixed medical and ND-affirming register; none was distinctively calibrated for ND-affirming communication.

## 4. Discussion

### 4.1. Principal Findings

This study provides one of the earliest systematic comparative evaluations of LLM quality for ASD caregiver information in a Middle Eastern context, and it is the first to apply a validated multi-dimensional quality framework (PEMAT-P, readability, ND-affirming language) to this domain in the Arab world. The results reveal a differentiated profile in which no single platform excelled across all dimensions. Gemini was the most scientifically accurate; ChatGPT was the most understandable and readable; DeepSeek was the most actionable. All three platforms failed to meet AHRQ actionability benchmarks, and all generated text at reading levels substantially above those recommended for patient education materials. These findings collectively indicate that the current generation of LLMs requires significant adaptation before they can be recommended as standalone ASD health information tools for lay caregivers.

### 4.2. Scientific Accuracy: Strengths and Systematic Blind Spots

The predominantly high accuracy of all three LLMs is broadly consistent with comparable evaluations of LLM performance in pediatric conditions. Yan et al. (2025) reported that LLMs generated acceptable information on Kawasaki disease in over 80% of responses, and the present study identified a similar ceiling pattern across most ASD questions [29]. The significant accuracy advantage of Gemini over DeepSeek (r = −0.37) and the systematic failure of all three LLMs on questions about Saudi-specific epidemiology (Q03), familial genetic risk (Q07), and treatment cost (Q17) reflect two structural limitations: training data imbalances favoring Western health contexts, and the inherent context-specificity of information in domains—such as treatment economics and regional service availability—where aggregate English-language training data is systematically unrepresentative of the Saudi reality [14,30]. These failures are not incidental but structural and cannot be resolved through prompt engineering alone.

### 4.3. Understandability and the Structural Advantage of ChatGPT

ChatGPT’s significantly superior PEMAT-P understandability relative to Gemini (r = 0.55, large effect) reflects its consistent structural formatting—organized headers, chunked paragraphs, and visual cues—that directly correspond to PEMAT-P criteria for plain-language health communication. These formatting choices align with evidence that structural clarity reduces cognitive load and improves comprehension in emotionally stressed lay audiences [31]. We note, however, that this formatting distinctiveness may also have compromised rater blinding, as experienced raters could plausibly recognize model identity from stylistic cues rather than evaluating content alone. This possibility limits the strength of causal claims linking formatting to ChatGPT’s performance advantage. Gemini’s lower and more variable understandability—reflected also in its poor inter-rater reliability (ICC = 0.446)—suggests a tendency toward discursive, encyclopedic prose that sacrifices accessibility for completeness. The co-occurrence of understandability and accuracy failures on the same question domains (notably Q17) supports the interpretation that LLM weaknesses cluster around contextually unfamiliar or geographically specific topics, rather than being randomly distributed across the question bank.

### 4.4. Actionability: A Pervasive and Clinically Urgent Deficit

The universal failure to meet the 80% PEMAT-P actionability benchmark is the most consistent and clinically consequential finding of this study. Actionability assesses whether a health resource tells readers what to do, breaks actions into explicit, manageable steps, and provides tangible tools, such as checklists or plans. LLMs are generative text models optimized to produce informative, descriptive content; they are not inherently structured to produce the prescriptive, step-by-step guidance that actionability instruments require. DeepSeek’s superior actionability (r = 0.59 vs. Gemini) suggests that the propensity to produce structured action lists may be trainable and/or amenable to prompt engineering—though our design manipulated neither variable, making this a hypothesis for future experimental testing, not a conclusion. This finding converges with evidence from broader health communication research demonstrating that actionability is among the most consistently deficient dimensions of web-based health content [32] and reinforces that LLMs currently replicate—rather than resolve—this longstanding limitation. Given that caregiver actionability directly influences daily behavioral management and intervention outcomes in ASD [33,34], the actionability deficit identified here represents a public health concern requiring urgent attention.

### 4.5. Readability: Consistently Exceeding Acceptable Limits

The large effect sizes (d > 1.0 for all LLMs on both FKGL and SMOG) confirm that the gap between actual LLM reading levels and patient education benchmarks is not trivial. Even ChatGPT, the most readable platform evaluated, requires approximately a 9th-grade reading level on average; Gemini’s mean SMOG of 12.50 corresponds to post-secondary education. These findings extend and confirm prior evidence of high reading levels in LLM-generated medical content [29,35] and demonstrate that parent-friendly prompting alone is insufficient to produce responses at appropriate reading levels for general caregiver audiences. Plain-language calibration appears to require structural interventions at the model or post-generation level: system-level instruction-tuning on plain-language health communication corpora, automated readability feedback loops, or clinical editorial review.

### 4.6. Neurodiversity-Affirming Language: An Unrealized Standard

The absence of significant differences between LLMs on ND-affirming language (*p* = 0.838) reflects a shared baseline limitation: all three LLMs defaulted to a mixed medical and ND-affirming register rather than consistently adopting the strengths-based, identity-affirming communication that is increasingly recognized as a standard of quality in ASD caregiver education [36,37]. Language framing in health communication is not a stylistic preference but a clinically material choice: deficit-based framing is associated with poorer parental adaptation and reduced engagement with interventions [38,39]. The consistent failure of all three LLMs on this dimension suggests that ND-affirming communication principles have not been systematically incorporated into the alignment of any of the evaluated models. An important internal validity caveat could arise from the study prompt (“a child who is possibly suffering from autism”), which used deficit-based framing, inconsistent with the ND-affirming standard being evaluated. This may have primed model responses toward a medicalized register, potentially contributing to the uniformly mixed ND-affirming scores. Future studies should test prompt sensitivity using neutral or affirming variants.

### 4.7. Clinical and Policy Implications and Practical Recommendations

For clinical practice in Saudi Arabia and comparable settings, three guidance principles emerge. First, for general factual information on ASD diagnosis, etiology, and prognosis, all three LLMs perform adequately on most questions; Gemini is the most reliably accurate. Caregivers should use LLMs for general explanatory content only, and should verify all Saudi-specific information (epidemiology, costs, services) with their healthcare team. Second, for practical daily management guidance, DeepSeek currently provides the most actionable responses, though all three fall far below acceptable standards for standalone patient education. Third, no available LLM generates ASD information at a reading level appropriate for general lay audiences; clinical mediation remains essential, particularly for families with limited formal education or limited English proficiency. For Saudi-specific questions—including local epidemiology, cultural attitudes toward ASD, and the cost and availability of Saudi health services—all three LLMs should be used with explicit caution and awareness of their structural limitations in non-Western contexts. Clinicians and healthcare providers should proactively provide curated, validated ASD resources in Arabic (e.g., Saudi Autism Society materials, MHRSD guidance) as counterweights to uncritical LLM use. They should also advise caregivers to use LLMs selectively as plain-language explainers of well-established content (what is ASD, basic diagnostic features) while avoiding reliance on them for actionable management guidance or locally specific information.

At the policy level, this study demonstrates the feasibility of rigorous, reproducible multi-dimensional LLM quality evaluation using validated instruments. Health ministries and digital health regulators should adopt such frameworks for pre-deployment and ongoing quality assurance of LLMs marketed or used for patient education. Platform developers should be encouraged to incorporate readability and actionability standards; RAG (retrieval-augmented generation) architectures are additionally recommended to address the systematic factual failures on Saudi-specific epidemiology, treatment costs, and local service availability. RAG can ground LLM outputs in verified local clinical guidelines, Ministry of Health data, and curated ASD caregiver resources, substantially improving factual accuracy without full retraining [14] and incorporating ND-affirming communication principles—into model alignment processes.

### 4.8. Limitations

The 24-question bank, while clinically validated, does not cover all ASD caregiver concern domains; questions about late diagnosis in women and girls [40], early screening in primary care [41], and psychiatric comorbidity management [42] were not included. The question bank was developed and validated by clinical experts only; no formal Delphi process or caregiver/parent panel was used. Caregiver priorities and phrasing preferences may differ from those of clinicians, and future iterations of this evaluation framework should include caregiver co-design, ideally through a multi-round Delphi process, to ensure content and face validity from the end-user perspective. The study was not pre-registered, and no prior analysis plan was deposited before data collection. Responses were generated in English over a single two-month window and may not generalize to Arabic-language outputs or future model versions. Arabic-language evaluation is a critical priority, as many Saudi Arabian ASD caregivers search in Arabic or use bilingual prompts, and LLM competence in Arabic language ASD discourse remains unevaluated. All conclusions should be treated as preliminary from an Arabic context perspective. The rater panel, while multi-disciplinary, did not include parent or caregiver raters. This is a primary limitation as experts’ ratings may not accurately reflect lay caregivers’ experience of understandability, actionability, or cultural appropriateness. The single-institution setting and expert panel composition may limit the generalizability of the question prioritization process. The lack of API use and parameter control limits replicability, and results may differ under API conditions with deterministic settings. Rater blinding to model identity may have been imperfect as ChatGPT’s consistent use of structured headers and numbered lists constitutes a recognizable stylistic signature. An experienced and frequent user of ChatGPT might recognize this pattern. Future studies should normalize formatting prior to blinded evaluation. The deficit-framed prompt “possibly suffering from autism” may have biased RQ4 results. Consumer interface access prevented parameter control; single-response sampling limits inference about output variability. Future studies should use API access with fixed random seeds or multiple sampling to assess response stability. This study did not employ system-role assignments, prompt optimization, or deterministic decoding parameters (e.g., low temperature, top-p/top-k tuning), any of which may improve model performance. Findings, therefore, reflect typical, unoptimized caregiver-style interactions and should not be generalized to API-based or prompt-engineered deployments. This study conducted multiple families of statistical comparisons. While Bonferroni correction was applied within each family, no correction was applied across the combined set of tests, raising the possibility of an inflated global Type I error rate. In addition, with n = 24 questions per model and a pronounced ceiling effect on various comparisons, the study was likely underpowered to detect true differences between models. Non-significant findings should therefore be interpreted with caution, and results should be viewed as exploratory rather than confirmatory. Raters recorded only the summed, denominator-adjusted percentage score for each PEMAT-P dimension per question and model; individual item-level ratings, including which specific items were scored Not Applicable, were not retained as a separate record. Consequently, item-level N/A frequencies cannot be reported for this dataset. Future applications of this evaluation framework will retain item-level scoring records so that N/A frequencies and other item-level patterns can be reported and audited.

## 5. Conclusions

No single LLM evaluated in this study meets the combined criteria for accurate, readable, actionable, and ND-affirming health information for ASD caregivers. Gemini, ChatGPT, and DeepSeek present distinct and complementary strengths, yet their persistent deficits in readability and actionability suggest that, in their current form, these LLMs are best positioned to complement rather than replace clinician-mediated caregiver education, and are not yet ready for standalone, unmediated use. The above conclusions apply specifically to Gemini 1.5 Pro, GPT-4o, and DeepSeek-V3, English outputs, and the 2-month window (April–May 2025) in the Saudi ASD caregiver context, and should not be extrapolated beyond these parameters without empirical validation. The generalizability of these findings to the broader Saudi ASD caregiver context requires further empirical validation. Future directions should include Arabic-language evaluation, structured prompt engineering and RAG-based interventions to improve readability and actionability, and co-designed evaluation frameworks developed with the Saudi ASD caregiver community. Until purpose-built, culturally calibrated, and clinically validated LLM tools are available, clinical guidance on their selective and critical use remains essential.

## Figures and Tables

**Table 1:** Inter-rater reliability [ICC (2, 1), two-way random, absolute agreement] across three raters (n = 24 questions per LLM).

Criterion	LLM	ICC (2, 1)	95% CI	Interpretation
Accuracy	Gemini	1.000	[1.000, 1.000]	Excellent
	ChatGPT	1.000	[1.000, 1.000]	Excellent
	DeepSeek	0.631	[0.404, 0.804]	Moderate
PEMAT-P Understandability	Gemini	0.446	[0.012, 0.765]	Poor
	ChatGPT	0.947	[0.897, 0.975]	Excellent
	DeepSeek	0.616	[0.102, 0.851]	Moderate
PEMAT-P Actionability	Gemini	0.839	[0.704, 0.922]	Good
	ChatGPT	0.948	[0.900, 0.975]	Excellent
	DeepSeek	0.719	[0.486, 0.862]	Moderate
Language	Gemini	0.635	[0.419, 0.804]	Moderate
	ChatGPT	0.777	[0.617, 0.887]	Good
	DeepSeek	0.912	[0.837, 0.958]	Excellent

Benchmarks: Excellent > 0.90, Good 0.75–0.90, Moderate 0.50–0.74, Poor < 0.50.

**Table 2:** Descriptive statistics for all outcome measures by LLM across 24 questions.

Outcome	LLM	Mean	SD	Median	Min	Max
Accuracy (1–3)	Gemini	2.96	0.20	3.00	2.00	3.00
	ChatGPT	2.79	0.51	3.00	1.00	3.00
	DeepSeek	2.64	0.47	3.00	1.67	3.00
ND-Affirming Language (1–3)	Gemini	2.12	0.29	2.00	2.00	3.00
	ChatGPT	2.14	0.32	2.00	2.00	3.00
	DeepSeek	2.21	0.55	2.00	1.00	3.00
Understandability (%)	Gemini	79.50	4.90	80.53	63.90	88.90
	ChatGPT	81.94	11.05	83.30	47.23	91.70
	DeepSeek	81.36	7.02	86.10	63.90	91.70
Actionability (%)	Gemini	51.11	11.06	46.67	40.00	80.00
	ChatGPT	56.11	17.57	60.00	20.00	80.00
	DeepSeek	65.83	13.09	66.67	40.00	80.00
FKGL	Gemini	10.37	2.51	10.30	5.40	14.80
	ChatGPT	7.86	1.86	8.30	4.70	11.40
	DeepSeek	9.06	1.67	9.00	4.90	12.90
SMOG	Gemini	12.50	1.94	12.30	9.30	16.10
	ChatGPT	9.97	1.39	10.15	7.20	12.50
	DeepSeek	10.78	1.25	10.95	8.10	14.20

Understandability and actionability are expressed as % of maximum possible PEMAT-P score. ND-Affirming = Neurodiversity-Affirming; FKGL = Flesch–Kincaid Grade Level; SMOG = Simple Measure of Gobbledygook. Recommended benchmarks: FKGL ≤ 6 (AMA/NIH); SMOG ≤ 8 (NIH); understandability ≥ 80% (AHRQ); actionability ≥ 80% (AHRQ).

**Table 3:** Kruskal–Wallis H tests and Bonferroni-corrected Mann–Whitney U post hoc comparisons.

Outcome	H(2)	*p*	Gemini vs. ChatGPT	Gemini vs. DeepSeek	ChatGPT vs. DeepSeek
Accuracy	9.23	0.010 *	*p* = 0.161	*p* = 0.003, r = −0.37 **	*p* = 0.119
Language	0.35	0.838	*p* = 0.820	*p* = 0.718	*p* = 0.588
Understandability %	9.03	0.011 *	*p* < 0.001, r = 0.55 **	*p* = 0.120	*p* = 0.516
Actionability %	12.05	0.002 **	*p* = 0.191	*p* < 0.001, r = 0.59 **	*p* = 0.060
FKGL	13.56	0.001 **	*p* = 0.001, r = −0.57 **	*p* = 0.049 *	*p* = 0.023 *
SMOG	21.26	<0.001 ***	*p* < 0.001, r = −0.70 **	*p* = 0.001, r = −0.55 **	*p* = 0.038 *

Adjusted α = 0.017. r = rank-biserial correlation. * *p* < 0.05; ** *p* ≤ 0.017; *** *p* < 0.001. Negative r = the first LLM had higher scores.

## Data Availability

The verbatim LLM responses (72 total) would be available on reasonable request. The data are not publicly available due to institutional data security protocols.

## Supplementary Materials

The following supporting information can be downloaded at: https://www.mdpi.com/article/10.3390/healthcare14142137/s1, (S1) the full scoring rubrics for Criteria 1 (Scientific Accuracy and Information Quality) and; (S2a and 2b) the item-level PEMAT-P scoring instrument; (S3) Criteria 3 (Neurodiversity-Affirming Language), (S4) the mathematical formulae for the Flesch–Kincaid Grade Level (FKGL) and SMOG readability indices with interpretive benchmarks; (S5) Extended Table showing Exact *p*-values, Effect Sizes with 95% CI, and Power and (S6) Selected Questions by the consultants.

## Abbreviations

The following abbreviations are used in this manuscript:

AHRQ	Agency for Healthcare Research and Quality
AI	Artificial intelligence
ASD	Autism spectrum disorder
FKGL	Flesch–Kincaid Grade Level
LLMs	Large language models
ND	neurodiversity
PEMAT-P	Patient Education Materials Assessment Tool for Printable Materials
SMOG	Simple Measure of Gobbledygook
