# Supplementary material for: LLMs in Medical Education for Autism Caregivers: A Comparative Evaluation of Accuracy, Readability, Actionability, and Neurodiversity-Affirming Language
Source: Healthcare (Basel). 2026 Jul 16;14(14):2137. doi: 10.3390/healthcare14142137 (PMC13409864; doi:10.3390/healthcare14142137)
Supplement: Supplementary file 1 [file healthcare-14-02137-s001.zip › healthcare-4406988-supplementary.pdf]

Supplementary Material file S1

Evaluation Criteria, Scoring Rubrics, Readability Formulae, and Questions Selected by Consultants

This supplementary file provides: (S1) the full scoring rubrics for Criteria 1 (Scientific Accuracy) and; (S2a and 2b) the item-level PEMAT-P scoring instrument; (S3) Criteria 3 (Neurodiversity-Affirming Language), (S4) the mathematical formulae for the Flesch–Kincaid Grade Level (FKGL) and SMOG readability indices with interpretive benchmarks; (S5) Extended Table showing Exact p-values, Effect Sizes with 95% CI, and Power and (S6) Selected Questions by the consultants.

S1. Scoring Rubrics for Criteria 1

S1.1 Criteria 1: Scientific Accuracy and Information Quality

Responses were rated on the following three-point ordinal scale, adapted from the Information Quality Grading framework [27]:

Supplementary Table S1.1. Scoring rubric for Criteria 1 (Scientific Accuracy and Information Quality).

Score	Label	Criterion
**3**	Completely correct, clear, and concise	The response is factually accurate and consistent with current ASD clinical guidelines; clearly communicated for a lay audience; and appropriately concise without significant omissions or padding.
**2**	Partially correct, clear, or concise	The response contains a mixture of accurate and inaccurate information; or is accurate but omits clinically important detail; or is unnecessarily verbose such that key information is obscured.
**1**	Completely incorrect, unclear, or not concise	The response is factually erroneous, contradicts current ASD evidence or guidelines, is irrelevant to the question posed, or is so poorly structured as to be uninformative for the target audience.

S2. Criteria 2: PEMAT-P Scoring Instrument

The Patient Education Materials Assessment Tool for Printable Materials (PEMAT-P) was developed and validated by the Agency for Healthcare Research and Quality (AHRQ) [26]. Items are scored dichotomously: Agree = 1, Disagree = 0, Not Applicable = excluded from the

denominator. Scores for each subscale are summed and divided by the total applicable items, then multiplied by 100 to yield a percentage. Materials scoring ≥80% are considered acceptable for health education purposes.

Supplementary Table S2.2a Understandability Items (Items 1–12)

#	Item	Response Options	Domain
1	The material makes its purpose completely evident.	Disagree=0, Agree=1	Content
2	The material does not include information or content that distracts from its purpose.	Disagree=0, Agree=1	Content
3	The material uses common, everyday language.	Disagree=0, Agree=1	Word Choice & Style
4	Medical terms are used only to familiarise the audience with the terms; when used, they are defined.	Disagree=0, Agree=1	Word Choice & Style
5	The material uses the active voice.	Disagree=0, Agree=1	Word Choice & Style
6	Numbers appearing in the material are clear and easy to understand.	Disagree=0, Agree=1, N/A	Use of Numbers
7	The material does not expect the user to perform calculations.	Disagree=0, Agree=1	Use of Numbers
8	The material breaks or 'chunks' information into short sections.	Disagree=0, Agree=1, N/A	Organisation
9	The material's sections have informative headers.	Disagree=0, Agree=1, N/A	Organisation
10	The material presents information in a logical sequence.	Disagree=0, Agree=1	Organisation
11	The material provides a summary.	Disagree=0, Agree=1, N/A	Organisation
12	The material uses visual cues (e.g., arrows, boxes, bullets, bold, larger font) to draw attention to key points.	Disagree=0, Agree=1, N/A	Layout & Design

PEMAT-P Understandability items (items 1–12), response options, and domain classification [26].

Supplementary Table S2.2b Actionability Items (Items 20–24)

#	Item	Response Options
20	The material clearly identifies at least one action the user can take.	Disagree=0, Agree=1
21	The material addresses the user directly when describing actions.	Disagree=0, Agree=1
22	The material breaks down any action into manageable, explicit steps.	Disagree=0, Agree=1
23	The material provides a tangible tool (e.g., menu planners, checklists) whenever it could help the user take action.	Disagree=0, Agree=1
24	The material provides simple instructions or examples of how to perform calculations.	Disagree=0, Agree=1, N/A

PEMAT-P Actionability items (items 20–24) and response options (Shoemaker et al., 2013).

S3.1 Criteria 3: Neurodiversity-Affirming Language

Responses were rated on the following three-point ordinal scale assessing the degree to which language was neurodiversity-affirming (ND-affirming) versus deficit-based and medicalised:

Supplementary Table S3.1 Scoring rubric for Criteria 3 (Neurodiversity-Affirming Language [27,28].

Score	Label	Criterion
**1**	Predominantly medical language	Response employs clinical or pathologising terminology throughout (e.g., ‘suffering from autism’, ‘abnormal behaviour’, ‘disorder’ as primary framing). No strengths-based or identity-affirming language is present.
**2**	Mixed language	Response combines medical or clinical terminology with accessible, person-first or identity-first language. Some neurodiversity-affirming framing is evident but does not predominate.

**3**	Predominantly ND-affirming language	Response consistently employs strengths-based, accessible language that affirms the lived experience of autistic individuals and their families (e.g., ‘different, not broken’; ‘different way the brain works’; celebrating individual strengths alongside challenges).

S4. Criteria 4: Readability Formulae and Interpretive Benchmarks

S4.1 Flesch–Kincaid Grade Level (FKGL)

The FKGL is calculated as:

FKGL = 0.39 × (Total Words / Total Sentences) + 11.8 × (Total Syllables / Total Words) − 15.59

The FKGL estimates the United States school grade level required to comprehend the text, where a score of 8 corresponds to an 8th-grade reading level. A score of ≤6 is recommended by the American Medical Association (AMA) and the National Institutes of Health (NIH) for health education materials targeting general adult audiences.

S4.2 SMOG Index (Simple Measure of Gobbledygook)

The SMOG index is calculated as:

SMOG = 3 + √(Polysyllabic Word Count × [30 / Sentence Count])

A polysyllabic word is defined as a word containing three or more syllables. The SMOG index is specifically validated for health education materials and tends to produce more conservative (higher) grade-level estimates than FKGL; it is accordingly the preferred readability instrument in health literacy research. An SMOG score of ≤8 is considered indicative of health material that is accessible to general adult audiences.

S4.3 Interpretive Benchmarks for FKGL and SMOG readability indices

**Instrument**	**Score / Grade**	**Readability Level**	**Recommended Threshold**
FKGL	≤6	Accessible for general adult health education	AMA / NIH: ≤6th grade
FKGL	7–8	Some difficulty for low-literacy adults	Acceptable with caution

FKGL	≥9	Difficult for lay audience	Exceeds recommended threshold
SMOG	≤8	Accessible for general adult health education	NIH / Health literacy standard
SMOG	9–12	Difficult for lay audience	Exceeds recommended threshold
SMOG	>12	Very difficult; graduate-level reading	Unacceptable for patient education

AMA = American Medical Association; NIH = National Institutes of Health.

S5. Extended Table showing Exact p-values, Effect Sizes with 95% CI, and Power

Outcome	Comparison	Statistic	Effect size (95% CI)	Exact p	Power (α=.05)	Power (αadj=.017)
Accuracy	Omnibus (Kruskal–Wallis)	H(2)=9.23	ε^2=0.105 [0.004, 0.309]	0.010	0.83	0.68
	Gemini vs ChatGPT	U=324.5	r=-0.127[-0.293, 0.042]	0.161	0.27	0.07
	Gemini vs DeepSeek	U=394.0	r=-0.368[-0.583, -0.153]	0.003	0.90	0.78
	ChatGPT vs DeepSeek	U=349.0	r=-0.212[-0.465, 0.056]	0.119	0.32	0.19
Language	Omnibus (Kruskal–Wallis)	H(2)=0.35	ε^2=-0.024[-0.028, 0.097]	0.838	0.08	0.03
	Gemini vs ChatGPT	U=296.0	r=-0.028[-0.250, 0.188]	0.820	0.05	0.02
	Gemini vs DeepSeek	U=273.5	r=0.050 [-0.214, 0.312]	0.718	0.07	0.02
	ChatGPT vs DeepSeek	U=267.0	r=0.073 [-0.186, 0.332]	0.588	0.09	0.03
Understandability	Omnibus (Kruskal–Wallis)	H(2)=9.03	ε^2=0.102 [0.003, 0.314]	0.011	0.82	0.68
	Gemini vs ChatGPT	U=129.0	r=0.552 [0.248, 0.839]	<0.001	0.93	0.87
	Gemini vs DeepSeek	U=216.0	r=0.250 [-0.083, 0.580]	0.120	0.38	0.24
	ChatGPT vs DeepSeek	U=319.5	r=-0.109[-0.425, 0.247]	0.516	0.11	0.05
Actionability	Omnibus (Kruskal–Wallis)	H(2)=12.05	ε^2=0.146 [0.027, 0.348]	0.002	0.93	0.84
	Gemini vs ChatGPT	U=227.0	r=0.212 [-0.097, 0.519]	0.191	0.28	0.15
	Gemini vs DeepSeek	U=119.5	r=0.585 [0.321, 0.814]	<0.001	0.98	0.93
	ChatGPT vs DeepSeek	U=198.0	r=0.312 [0.007, 0.613]	0.060	0.49	0.32

FKGL	Omnibus (Kruskal–Wallis)	H(2)=13.56	ε^2=0.167 [0.034, 0.394]	0.001	0.94	0.87
	Gemini vs ChatGPT	U=451.0	r=-0.566[-0.797, -0.292]	<0.001	0.95	0.89
	Gemini vs DeepSeek	U=384.0	r=-0.333[-0.637, -0.019]	0.049	0.53	0.35
	ChatGPT vs DeepSeek	U=177.0	r=0.385 [0.066, 0.679]	0.023	0.65	0.45
SMOG	Omnibus (Kruskal–Wallis)	H(2)=21.26	ε^2=0.279 [0.105, 0.508]	<0.001	1.00	0.99
	Gemini vs ChatGPT	U=489.0	r=-0.698[-0.892, -0.458]	<0.001	1.00	0.99
	Gemini vs DeepSeek	U=446.0	r=-0.549[-0.806, -0.266]	0.001	0.93	0.85
	ChatGPT vs DeepSeek	U=187.0	r=0.351 [0.031, 0.642]	0.038	0.56	0.37

S6. Selected Questions by the consultants

1. What is Autism?
2. How do we know that the hyperactivity of our child is related to Autism?
3. How common is Autism in children?
4. What are some common features seen in Autism
5. How is Autism diagnosed?
6. Who is the best kind of doctor to diagnose our child, possibly suffering from Autism?
7. Will Autism run in our family?
8. How will Autism cause changes in the brain function of our child?
9. How will Autism affect the growth of our child?
10. What are the common treatment options for Autism?
11. How long does our child with Autism have to take medicine?
12. How can we help our child prevent psychological problems caused by Autism?
13. How can we manage our child with Autism for the long term?
14. During the recovery of our child with Autism, do we need to pay special attention to anything?
15. Can our child with Autism go to an ordinary school or a special care centre?
16. How to handle the bizarre behaviour of our child with Autism?
17. Is the treatment of Autism expensive?
18. Do children with Autism not socialize at all?
19. How to calm our child with Autism during tantrums or cries?
20. Can our child with Autism ever live an independent life?
21. How do we teach our child with Autism to do physical activities independently?
22. Do you have any advice for us as concerned parents, as we have too much to do?
23. Why does our child with Autism not make eye contact, smile and respond to our facial expressions or greetings?
24. Why does our child with Autism get upset by changes in routine or familiar surroundings?

References (are list in the manuscript)

26. Shoemaker, S. J., Wolf, M. S., & Brach, C. (2013). Patient education materials assessment tool for printable materials (PEMAT-P). Agency for Healthcare Research and Quality.

27. Bottema-Beutel, K., Kapp, S. K., Lester, J. N., Sasson, N. J., & Hand, B. N. (2021). Avoiding ableist language: Suggestions for autism researchers. Autism in Adulthood, 3(1), 18–29. https://doi.org/10.1089/aut.2020.0014

28. Kenny, L., Hattersley, C., Molins, B., Buckley, C., Povey, C., & Pellicano, E. (2016). Which terms should be used to describe autism? Perspectives from the UK autism community. Autism, 20(4), 442–462. https://doi.org/10.1177/1362361315588200

29. Yan, C., Li, Z., Liang, Y., Shao, S., Ma, F., Zhang, N., Li, B., Wang, C., & Zhou, K. (2025). Assessing large language models as assistive tools in medical consultations for Kawasaki disease. Frontiers in Artificial Intelligence, 8, 1571503. https://doi.org/10.3389/frai.2025.1571503
